# Genetic Variations in the Androgen Receptor Are Associated with Steroid Concentrations and Anthropometrics but Not with Muscle Mass in Healthy Young Men

**DOI:** 10.1371/journal.pone.0086235

**Published:** 2014-01-23

**Authors:** Hélène De Naeyer, Veerle Bogaert, Annelies De Spaey, Greet Roef, Sara Vandewalle, Wim Derave, Youri Taes, Jean-Marc Kaufman

**Affiliations:** 1 Department of Endocrinology, Ghent University Hospital, Ghent, Belgium; 2 Department of Movement and Sport Sciences, Ghent University, Ghent, Belgium; 3 Unit for Osteoporosis and Metabolic bone diseases, Ghent University Hospital, Ghent, Belgium; Children's National Medical Center, Washington, United States of America

## Abstract

**Objective:**

The relationship between serum testosterone (T) levels, muscle mass and muscle force in eugonadal men is incompletely understood. As polymorphisms in the androgen receptor (*AR*) gene cause differences in androgen sensitivity, no straightforward correlation can be observed between the interindividual variation in T levels and different phenotypes. Therefore, we aim to investigate the relationship between genetic variations in the *AR*, circulating androgens and muscle mass and function in young healthy male siblings.

**Design:**

677 men (25–45 years) were recruited in a cross-sectional, population-based sibling pair study.

**Methods:**

Relations between genetic variation in the *AR* gene (CAGn, GGNn, SNPs), sex steroid levels (by LC-MS/MS), body composition (by DXA), muscle cross-sectional area (CSA) (by pQCT), muscle force (isokinetic peak torque, grip strength) and anthropometrics were studied using linear mixed-effect modelling.

**Results:**

Muscle mass and force were highly heritable and related to age, physical activity, body composition and anthropometrics. Total T (TT) and free T (FT) levels were positively related to muscle CSA, whereas estradiol (E_2_) and free E_2_ (FE_2_) concentrations were negatively associated with muscle force. Subjects with longer CAG repeat length had higher circulating TT, FT, and higher E_2_ and FE_2_ concentrations. Weak associations with TT and FT were found for the rs5965433 and rs5919392 SNP in the *AR*, whereas no association between GGN repeat polymorphism and T concentrations were found. Arm span and 2D:4D finger length ratio were inversely associated, whereas muscle mass and force were not associated with the number of CAG repeats.

**Conclusions:**

Age, physical activity, body composition, sex steroid levels and anthropometrics are determinants of muscle mass and function in young men. Although the number of CAG repeats of the *AR* are related to sex steroid levels and anthropometrics, we have no evidence that these variations in the *AR* gene also affect muscle mass or function.

## Introduction

Skeletal muscle mass and function are highly heritable [1] and influenced by age, anthropometrics, sex steroid status and lifestyle-related factors [2]–[4]. The clinical relationship between androgens and muscle mass is well-described. Androgen deficiency (i.e. hypogonadism) leads to significant muscle loss and weakness [5], whereas testosterone (T) supplementation has dose-dependent anabolic effects [6], [7]. Moreover, impaired steroid production or low androgen sensitivity could interfere with normal bone development and closure of the epiphyseal growth plates at the end of puberty.

However, the interrelationship between T levels, muscle mass and muscle force in eugonadal men is less clear [8]. Serum T levels are maintained at appropriate levels by the hypothalamic-pituitary-gonadal feedback loop. In healthy men, a large interindividual variation in serum T levels exists [9]. This between-subjects variability in T levels has been related to environmental conditions such as age, body mass index and smoking [10], and is considerably influenced by genetic factors [11], [12]. The sensitivity to circulating T is determined in part by the transcriptional activity of the androgen receptor (*AR*). Polymorphisms in the *AR* gene have been described to alter this activity. We have previously shown that diminished androgen feedback, and consequently higher serum T concentrations, are associated with the CAG repeat length, and to a lesser extend with the GGN repeat length [9], [13]. Furthermore, some single nucleotide polymorphisms (SNP) in the *AR* gene, resulting in an altered binding with cofactors, have been linked with the androgen insensitivity syndrome (AIS) [14]–[16] and could therefore affect androgen action and circulating androgen levels.

In order to gain more insight into the between subject variation in muscle mass in young healthy men, we investigated the relationship between androgens and muscle mass and function, as well as the influence of genetic components. We hypothesized that genetic variations in the *AR*, causing differences in androgen sensitivity, contribute to the variation in muscle mass in young healthy men.

## Materials and Methods

### Ethics statement

The study protocol was conducted according to the Helsinki Declaration and was approved by the ethical committee of the Ghent University Hospital. All participants gave their written informed consent and questionnaires about previous illness and medication use were completed. Physical activity was scored using the questionnaire as proposed by Baecke *et al.* [17].

### Study design and population

This population-based cross-sectional study is part of a larger study, from which inclusion criteria and study design were described previously [18]. Participants were recruited from the population registries of 3 semi-rural to suburban communities around Ghent, Belgium. Men (n = 12446), 25–45 years of age were contacted by direct mailing, briefly describing the study purpose and asking if they had a brother within the same age range also willing to participate (maximal age difference between brothers was set at 12 yrs). The overall response rate was 30.2%. Finally, a sample of 768 young healthy men who fulfilled the primary inclusion criterion of having a brother within the same age range agreed to participate. After exclusions, 677 men in total were included in the study. Two hundred ninety six pairs of brothers (for a total of 592 men) were included in addition to 64 men as single participants, when their brother could not participate in the study; 19 men were included as third brother in a family and 2 as fourth brother. Exclusion criteria were defined as illnesses or medication use affecting body composition, hormone levels or bone metabolism.

### Body composition and muscle strength

Body weight and anthropometrics (arm span, hand and finger length) were measured in light indoor clothing without shoes. Sternum height was measured using a wall-mounted Harpenden stadiometer (Holtain, Crymych, UK). Lean and fat mass of the whole body were measured using dual-energy x-ray absorptiometry (DXA) with a Hologic QDR-4500A device (software version 11.2.1; Hologic, Bedford, MA, USA). Isokinetic peak torque of biceps and quadriceps muscles was assessed at the dominant limbs using an isokinetic dynamometer (Biodex, New York, NY, USA). Grip strength at the dominant hand was measured using an adjustable hand-held standard grip device (JAMAR hand dynamometer; Sammons & Preston, Bolingbrook, IL, USA). Their maximum performance was assumed to best reflect the current status and the history of their musculoskeletal adaptation.

### Cross-sectional muscle area

A peripheral quantitative computed tomography (pQCT) device (XCT-2000, software version 5.4; Stratec Medizintechnik, Pforzheim, Germany) was used to scan the dominant leg (tibia) and forearm (radius). Muscle cross-sectional area (CSA) was estimated using a threshold below water equivalent linear attenuation set at 0.22/cm. This threshold eliminated skin and fat mass with lower linear attenuation in the cross-sectional slice. From the remaining area, bone area was subtracted, revealing the muscle at its maximum CSA.

### Biochemical determinations

Venous blood samples were obtained between 08:00 and 10:00 AM after overnight fasting. All serum samples were stored at −80°C until batch analysis. Serum total testosterone (TT) and estradiol (E_2_) levels were determined by liquid chromatography tandem mass spectrometry (LC-MS/MS) (AB Sciex 5500 triple-quadrupole mass spectrometer; AB Sciex, Toronto, Canada). Serum limit of quantification was <0.5 pg/mL (1.9 pmol/L) for E_2_ and 1.2 ng/dL for T. The interassay coefficients of variation (CV) were 4.0% at 21 pg/mL (77 pmol/L) for E_2_, and 8.3% at 36.7 ng/dL and 3.1% at 307.8 ng/dL for T [19]. Commercial radioimmunoassays were used to determine serum levels of sex hormone binding globulin (SHBG) (Orion Diagnostica, Espoo, Finland), luteinizing hormone (LH) and follicle-stimulating hormone (FSH) (ECLIA; Roche Diagnostics, Mannheim, Germany). Free testosterone (FT) and free estradiol (FE_2_) concentrations were calculated from serum TT, E_2_, SHBG and albumin concentrations using a previously validated equation derived from the mass action law [20], [21].

### Genotyping of the androgen receptor

Genomic DNA was extracted from EDTA-treated blood using a commercial kit (Puregene kit; Gentra Systems, Minneapolis, MN, USA). The CAG and GGN repeats were determined as previously described [13].

Genotyping data for the AR gene for the Caucasian CEPH population was downloaded from the International Haplotype Mapping Project web site (http://www.hapmap.org) and the data was incorporated into the Haploview program [22]. The tagger function within Haploview was used to assign Tag SNPs. The tagging SNPs were chosen, by aggressive tagging (use 2- or 3-marker haplotypes), to capture the variations within the gene and the surrounding area with minor allele frequency (MAF) 0.01 and a minimum r^2^ of 0.80 (for their location and the SNPs which they tag). For the SNP analyses, SNPlex [23] was carried out on fragmented gDNA at a final concentration of 25 ng/µl (total volume of 9 µl). Samples were run on an ABI 3730xl DNA Analyzer (Applied Biosystems, Foster City, CA, USA) and data were analysed using Gene Mapper v. 3.7 software (Applied Biosystems). Genotype analysis was performed based on the SNPlex_Rules_3730 method following the factory default rules. Missing genotypes in the SNPlex analysis were obtained using TagMan Pre-Designed SNP Genotyping Assays® (Applied Biosystems) which were run on the StepOne System (Applied Biosystems). In total, 5 SNPs of the AR gene were genotyped.

### Statistics

Descriptives are expressed as mean ± standard deviation or median [1^st^–3^rd^ quartile] when criteria for normality were not fulfilled (Kolmogorov-Smirnov) and variables were log-transformed in subsequent linear models. Linear mixed-effects modelling with random intercepts and a simple residual correlation structure was used to study the effect of anthropometrics, sex steroid concentrations and genetic variations in the *AR* on muscle mass and function, with adjustment for the confounding effect of age, adult height and weight or fat mass and taking into account the interdependence of measurements between brothers. Parameters of fixed effects were estimated via restricted maximum likelihood estimation and reported as estimates of effect size (β) with their respective standard error. A sample size of 677 subjects allowed us a 81% power to detect a minimum effect size of 0.01 at a two-sided significance level of 5%. Validity of the models was assessed by exploring normality of distribution of the residuals. SNPs were considered as a categorical variable, whereas CAG and GGN lengths were analysed as continuous variables for assessing association, and as categorical variable (quartiles) with groups compared by one-way analysis of variance (ANOVA). Associations were considered significant at p-values less than 0.05. Statistical analyses were performed using S-Plus 7.0 (Insightful, Seattle, WA, USA). The polygenic program in SOLAR 2.0 (Southwest Foundation for Biomedical Research, San Antonio, TX, USA) was used to estimate heritability, using a variance component model.

## Results

### Study population and characteristics

Six hundred seventy seven subjects with a mean age of 34.5±5.5 years are included in the study. Mean height is 1.79±0.06 m and mean weight 81.4±11.8 kg, with a body mass index of 25.3±3.5 kg/m^2^. Body composition and muscle function parameters are given in Table 1.

**Table 1:** General characteristics and hormone concentrations of all study participants (n = 677).

	Mean ± SD
**Age (yr)**	34.5±5.5
**Weight (kg)**	81.4±11.8
**Height (m)**	1.79±0.06
**BMI (kg/m^2^)**	25.3±3.5
**Testosterone (ng/dL)**	579 [467.0–703.8]
**Free testosterone (ng/dL)**	14.2 [11.9–17.0]
**Estradiol (ng/dL)**	2.12 [1.67–2.57]
**Free estradiol (ng/dL)**	0.04 [0.03–0.05]
**SHBG (nmol/L)**	23 [18.4–29.7]
**LH (U/L)**	4.3 [3.1–5.5]
**FSH (U/L)**	3.8 [2.7–5.4]
**Whole body lean mass (kg)**	62.2±6.6
**Whole body fat mass (kg)**	16.4±6.4
**Radius 66% muscle area (cm^2^)**	45.2±5.9
**Tibia 66% muscle area (cm^2^)**	82.6±11.1
**Grip strength (kg)**	51.7±8.0
**Biceps force (Nm)**	57.3±10.5
**Quadriceps force (Nm)**	203±42
**Arm span (cm)**	182.7±7.3
**Hand length (cm)**	20.5±1.0
**Digit 2 finger length (cm)**	7.4±0.5
**Digit 4 finger length (cm)**	7.6±0.5
**Sternum height (cm)**	61.5±2.7

Non-Gaussian distribution: data presented as median [1^st^–3^rd^ quartile]. Free testosterone and free estradiol serum concentrations were calculated using previously validated equations [20], [21].

As expected, the level of physical activity was associated with muscle mass. Biceps force was positively associated with the level of physical activity during work (β : 0.18±0.03; p<0.0001) but not related to physical activity during sports (p = 0.96), whereas quadriceps force was related to sports (β : 0.11±0.04; p = 0.004) and not to physical activity during work (p = 0.52), independent from age, height and weight.

### Age, weight and height in relation to muscle mass and force

Both fat (β : 0.2±0.05 kg/y; p = 0.0001) and lean mass (β : 0.1±0.05 kg/y; p = 0.03) increased with age, as well as muscle CSA at the radius (β : 21 mm^2^/y±4; p<0.0001) and tibia (β : 32 mm^2^/y±8; p = 0.0001), which remained positive after additional adjustment for height, physical activity level and body fat (radius: p<0.0001 and tibia: p = 0.004). With increasing age, lower limb muscle force indices slightly decreased after adjustment for height and weight (p = 0.02). Biceps muscle force and maximal grip strength were unrelated to age.

Whole body lean mass was positively associated with height (β : 0.22±0.02; p<0.0001) and weight (β : 0.78±0.02; p<0.0001). Also a close relationship between muscle CSA and weight (β : 0.54±0.03; p<0.0001 for radius, and β : 0.56±0.03; p<0.0001 for tibia) was found. Moreover, maximal grip strength and muscle force indices at upper (biceps) and lower limb (quadriceps) were all positively related to height (all p<0.0001) and weight (all p<0.001).

Whole body lean mass exhibited a strong positive association with muscle CSA and muscle function (all p<0.0001), whereas whole body fat mass was inversely related to muscle CSA at radius (p<0.0001) and grip strength and muscle force of biceps (p<0.001).

**Figure 1:**
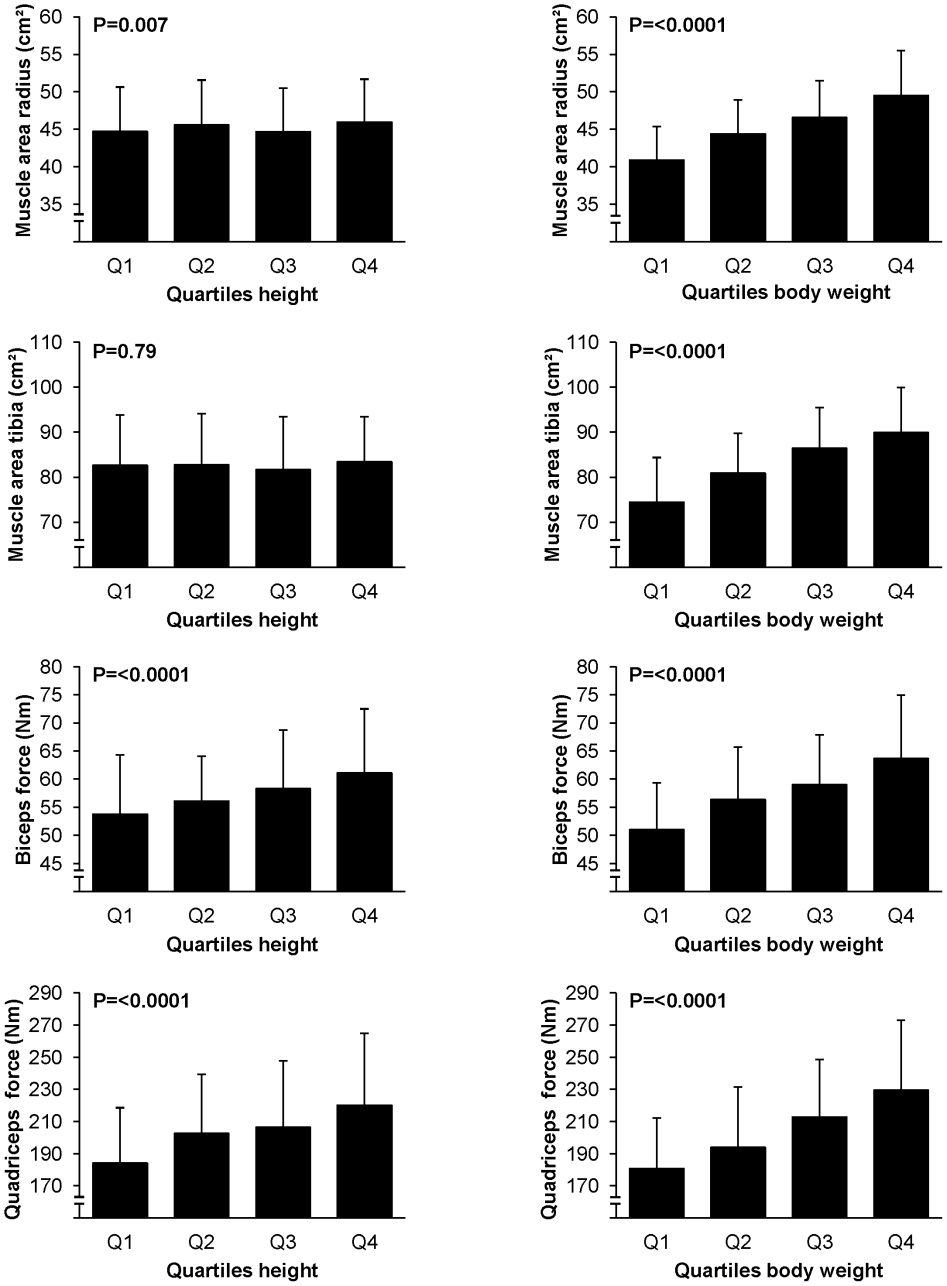
Muscle CSA and muscle force (grip, biceps and quadriceps) according to quartiles of height and weight. P-values result from ANOVA (overall difference between categories). Each bar represents the mean ± standard deviation (SD).

The relationship of muscle CSA and muscle force (grip, biceps and quadriceps) with height and weight are represented in Figure 1.

### Heritability of muscle mass and function

Table 2 illustrates the heritabilities of muscle mass and function parameters. All parameters are highly heritable (p<0.0001), with the highest h^2^ observed for whole body lean mass.

**Table 2:** Heritability estimates of selected muscle parameters.

	h^2^
**Whole body lean mass (kg)**	0.86±0.09
**Whole body fat mass (kg)**	0.73±0.10
**Radius 66% muscle area (mm^2^)**	0.67±0.10
**Tibia 66% muscle area (mm^2^)**	0.63±0.10
**Grip strength (kg)**	0.56±0.10
**Biceps force (Nm)**	0.76±0.10
**Quadriceps force (Nm)**	0.67±0.10

### Muscle mass and force in relation to anthropometric measurements

Whole body lean mass and muscle CSA at the radius were positively associated with arm span (β : 0.29±0.05; p<0.0001 and β : 0.31±0.07; p<0.0001 respectively) as well as with finger (p = 0.0001 to 0.04) and hand length (all p<0.0001) adjusted for height, weight and age. Fat mass was negatively associated with arm span (β : −0.23±0.03; p<0.0001). Moreover, biceps flexion and hand grip force were related to arm span (β : 0.46±0.06; p<0.0001 for biceps and β : 0.48±0.07; p<0.0001 for grip), even more strongly than to hand length (β : 0.32 to 0.34±0.05; p<0.0001 for biceps and β : 0.33±0.05; p<0.0001 for grip) and finger length (β : 0.19 to 0.24±0.04 ; p<0.0001 for biceps and β : 0.25 to 0.30±0.04; p<0.0001 for grip). Muscle force and muscle CSA were unrelated to sternum height (data not shown). All associations remained positive after additional adjustment for fat or lean mass.

### Sex steroids in relation to muscle mass and function

TT and FT concentrations were positively related to whole body lean mass (β : 0.07±0.02; p = 0.0002 and β : 0.08±0.02; p<0.0001 respectively) and inversely to fat mass (β : −0.07±0.02; p = 0.0001 and β : −0.08±0.02; p<0.0001 respectively), adjusted for age, weight and height. TT concentrations were positively related to muscle CSA at the tibia (β : 0.07±0.04; p = 0.04), and FT was positively associated with muscle CSA at the radius (β : 0.07±0.04; p = 0.03). E_2_ and FE_2_ concentrations were negatively associated with maximal grip strength (β : −0.08±0.04; p = 0.04 and β : −0.10±0.04; p = 0.007 respectively) and quadriceps force (β : −0.08±0.04; p = 0.02 and β : −0.11±0.04; p = 0.002 respectively), even after additional adjustment for T. No influence of TT or FT on muscle force was observed (data not shown). The 2D:4D finger length ratio and arm span were unrelated to circulating steroid concentrations (data not shown).

### Genetic variation in AR in relation to circulating sex steroids, anthropometrics and muscle mass and function

The influence of genetic variation in the *AR* on circulating gonadal steroids, body composition and muscle function is shown in Table 3. The CAG repeat demonstrated a positive association with circulating TT and FT concentration, as well as with E_2_ and FE_2_ concentrations. Weak associations were found for the rs5965433 and rs5919392 polymorphisms in the *AR*. However, only the association between CAG repeat and TT and FT remained significant after Bonferroni correction. No associations between GGN repeat polymorphism and TT or FT concentrations, as determined by LC-MS/MS, were found.

**Table 3:** Androgen receptor polymorphisms in relation to circulating gonadal steroids and muscle parameters.

	CAG repeat	GGN repeat	rs17217069	rs5965433	rs5919392	rs6152	rs12011793
**Testosterone (ng/dL)**	**0.10±0.04 (p = 0.004)**	0.06±0.04 (p = 0.09)	0.44±0.26 (p = 0.10)	−0.28±0.14 (p = 0.05)	0.27±0.16 (p = 0.10)	0.16±0.11 (p = 0.12)	0.03±0.14 (p = 0.84)
**Free testosterone (ng/dL)**	**0.17±0.04 (p<0.0001)**	0.06±0.04 (p = 0.08)	0.41±0.28 (p = 0.14)	**−0.30±0.14 (p = 0.04)**	**0.35±0.17 (p = 0.04)**	0.19±0.11 (p = 0.08)	0.12±0.14 (p = 0.40)
**Estradiol (ng/dL)**	**0.08±0.04 (p = 0.05)**	0.07±0.04 (p = 0.07)	0.32±0.30 (p = 0.029)	0.07±0.15 (p = 0.65)	0.16±0.18 (p = 0.39)	0.11±0.12 (p = 0.36)	0.25±0.15 (p = 0.09)
**Free estradiol (ng/dL)**	**0.10±0.04 (p = 0.014)**	0.07±0.04 (p = 0.07)	0.29±0.29 (p = 0.33)	0.06±0.15 (p = 0.68)	0.17±0.18 (p = 0.35)	0.10±0.12 (p = 0.37)	0.26±0.15 (p = 0.08)
**SHBG (nmol/L)**	−0.05±0.04 (p = 0.21)	0.02±0.04 (p = 0.52)	0.40±0.27 (p = 0.14)	−0.13±0.14 (p = 0.35)	0.02±0.17 (p = 0.89)	0.04±0.11 (p = 0.69)	−0.14±0.14 (p = 0.31)
**LH (U/L)**	0.06±0.04 (p = 0.14)	0.05±0.04 (p = 0.23)	0.22±0.30 (p = 0.45)	−0.12±0.15 (p = 0.42)	−0.05±0.18 (p = 0.80)	0.23±0.12 (p = 0.05)	0.22±0.15 (p = 0.14)
**FSH (U/L)**	−0.07±0.04 (p = 0.07)	0.06±0.04 (p = 0.15)	−0.34±0.29 (p = 0.24)	0.29±0.15 (p = 0.05)	−0.12±0.18 (p = 0.49)	−0.04±0.11 (p = 0.70)	0.10±0.15 (p = 0.51)
**Whole body lean mass (kg)**	−0.004±0.017 (p = 0.80)	0.02±0.02 (p = 0.20)	−0.17±0.12 (p = 0.17)	−0.08±0.07 (p = 0.24)	0.10±0.08 (p = 0.22)	0.09±0.05 (p = 0.08)	0.10±0.06 (p = 0.11)
**Whole body fat mass (kg)**	−0.005±0.018 (p = 0.80)	−0.02±0.02 (p = 0.30)	0.23±0.13 (p = 0.08)	0.06±0.07 (p = 0.40)	−0.08±0.08 (p = 0.34)	−0.10±0.05 (p = 0.05)	−0.12±0.07 (p = 0.07)
**Radius 66% muscle area (mm^2^)**	−0.04±0.03 (p = 0.17)	0.04±0.03 (p = 0.22)	−0.03±0.24 (p = 0.89)	−0.07±0.12 (p = 0.57)	0.33±0.15 (p = 0.02)	0.15±0.09 (p = 0.11)	0.17±0.12 (p = 0.15)
**Tibia 66% muscle area (mm^2^)**	0.005±0.033 (p = 0.90)	0.01±0.03 (p = 0.75)	−0.05±0.24 (p = 0.84)	−0.11±0.12 (p = 0.36)	−0.07±0.15 (p = 0.66)	0.08±0.10 (p = 0.39)	0.07±0.12 (p = 0.59)
**Grip strength (kg)**	−0.02±0.04 (p = 0.53)	−0.002±0.036 (p = 0.97)	−0.004±0.271 (p = 0.99)	−0.002±0.140 (p = 0.99)	−0.02±0.17 (p = 0.90)	0.09±0.11 (p = 0.41)	0.14±0.14 (p = 0.32)
**Biceps force (Nm)**	−0.05±0.04 (p = 0.21)	0.03±0.03 (p = 0.42)	−0.09±0.27 (p = 0.73)	−0.18±0.13 (p = 0.17)	0.29±0.16 (p = 0.07)	0.04±0.10 (p = 0.69)	0.04±0.13 (p = 0.77)
**Quadriceps force (Nm)**	−0.02±0.04 (p = 0.51)	0.03±0.04 (p = 0.38)	−0.16±0.28 (p = 0.56)	−0.07±0.14 (p = 0.59)	0.19±0.16 (p = 0.24)	−0.007±0.104 (p = 0.95)	0.02±0.13 (p = 0.87)

Data are presented as standardized estimate ± SD (p-value). Results from mixed effects accounted for family structure and adjusted for age, height and weight.

**Figure 2:**
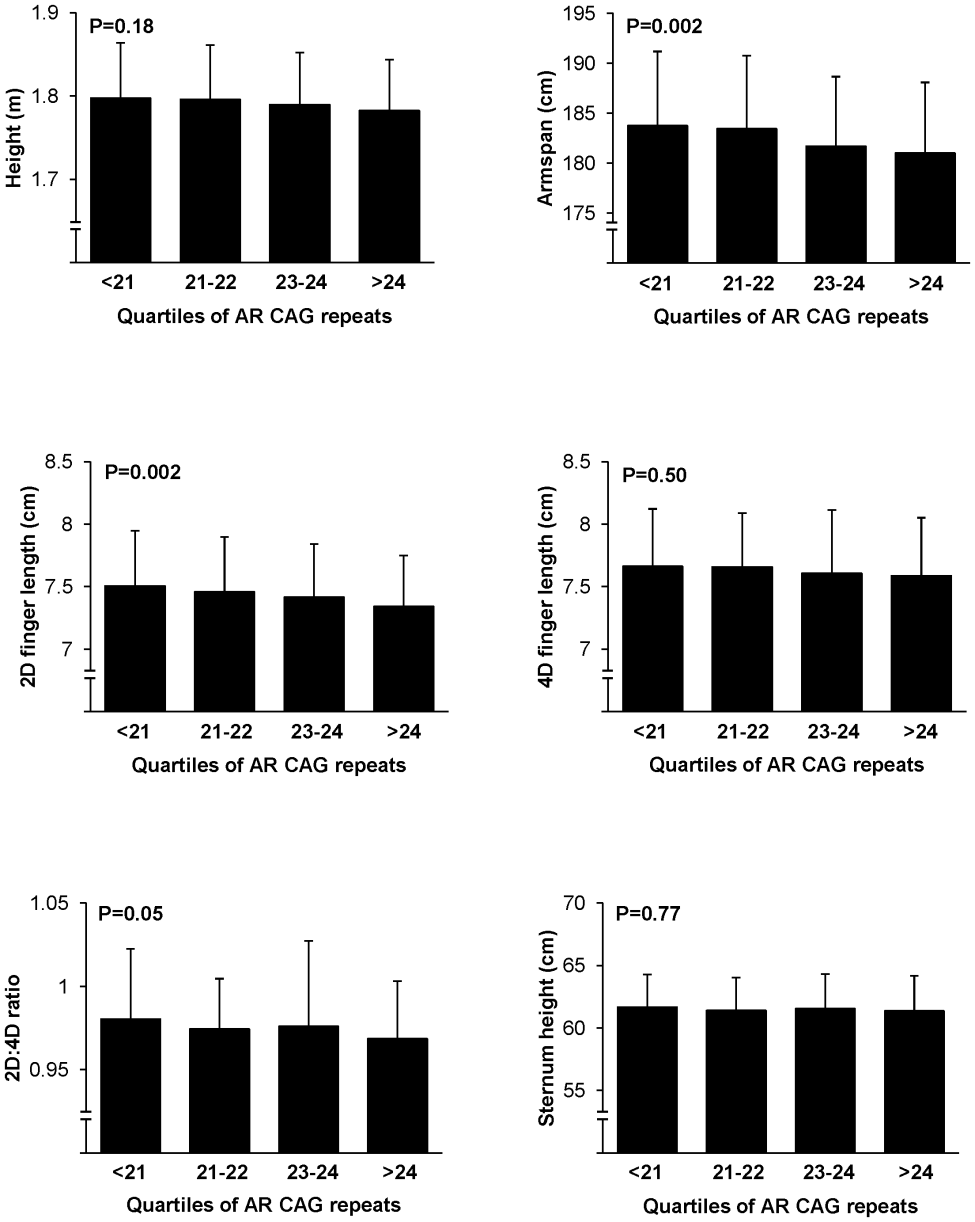
Anthropometrics according to quartiles of AR CAG repeat polymorphism. P-values result from ANOVA (overall difference between categories). Each bar represents the mean ± standard deviation (SD).

No consistent effects of the *AR* polymorphisms or CAG/GGN repeats were found on either body composition, muscle mass or muscle force (Table 3). Figure 2 illustrates the influence of the CAG repeat polymorphism on anthropometrics. Arm span was inversely associated with the number of CAG repeats (β : −0.09±0.02; p = 0.0001). Adult height (Figure 2), hand and digit 4 length (data not shown) were unrelated to CAG length, but digit 2 length at both left and right hand was inversely related to the CAG polymorphism (right β : −0.04±0.01; p = 0.0002 and left β : −0.04±0.01; p = 0.002 adjusted for age and height). From the 7 genetic variations analysed, only the CAG repeat length was found to be negatively related to the 2D:4D finger length ratio (right β : −0.05±0.01; p = 0.0006 and left β: −0.03±0.01; p = 0.01).

## Discussion

In this cross-sectional study we investigated the interrelation between androgen sensitivity, heritability, circulating sex steroids, anthropometrics and muscle mass and function in a cohort of young men. We observed that the number of CAG repeats is associated with TT, FT, E_2_ and FE_2_ levels, and the 2D:4D finger length ratio and arm span. In contrast with the observed associations with circulating sex steroids, these genetic variations in the *AR* did not influence muscle mass or function in this cohort of young healthy men.

Our results are in agreement with twin studies reporting that muscle mass and strength are highly heritable [1]. Some of the remaining variance in muscle mass might be explained by antropometry, which is also under genetic control [2]–[4]. Height and weight were closely related to lean mass in our study. As taller subjects have longer bones, it is reasonable that they have longer muscles and thus higher muscle mass. Biceps force and hand grip force were also found to be related with anthropometric measurements, demonstrating that the strength of an individual is strongly determined by its body size.

Age has also an influence on skeletal muscle mass and function [3]. However, few studies have examined the relationship between age and lean mass in (young) adults [24], [25]. In our study, we found a small but positive association between lean mass, muscle CSA and age. The association of age with grip and biceps force, and the small inverse relationship with quadriceps force supports the results of Janssen *et al.* [25] which state that the muscle strength of the upper body is preserved better with increasing age than the muscle strength of the lower body.

The alterations in body composition with aging are thought to be related to changes in sex steroid levels [26]. A loss of lean mass and an increase in fat mass are observed in elderly and hypogonadal men, whereas puberty in boys is associated with a remarkable gain in muscle mass [3], [5]. However, the clinical relationship between androgens and muscle mass for variations within the normal range is less clear. In this cohort of eugonadal men, we demonstrated that whole body lean mass and muscle CSA are positively associated with both TT and FT. It is noteworthy that physical activity was also positively associated with serum T concentrations, indicating a higher impact of physical activity on muscle mass in men with higher serum T levels. However, and in agreement with Folland *et al.* [8], further analysis revealed that neither TT nor FT had any relation with muscle strength.

As mentioned earlier, between-subject differences in serum T levels within the physiological range are related in part to differences in androgen sensitivity and hypothalamus-pituitary feedback setpoint [9]. Genetic variations in the *AR* gene, in particulary CAG repeat polymorphisms, have been associated with disorders linked to a reduced androgen activity [27]. We have previously shown that serum T levels are positively associated with the CAG and GGN repeat length in young, middle-aged and elderly men [9], [13]. This is in contrast with the present study, in which we did not find any correlation between TT or FT and the GGN repeat length. It is noteworthy that the subjects of the current study are partly overlapping (358 unrelated men i.e. a single representative of the nuclear families out of 677 men) with the cohort of young men published by Crabbe *et al.* [9] and Bogaert *et al.* [13]. However, the serum concentrations of T have been re-determined by a highly precise LC-MS/MS method, as these were previously determined using less specific commercial immunoassay kits. Reports on associations between the GGN repeat and AR function are limited and inconsistent, with one study describing a positive association in a cohort of men with prostate cancer [28], whereas another study in young men could not find an association between the GGN repeat and serum T levels [29].

Based on studies reporting mutations in the *AR* gene related to AIS [14]–[16] we further screened for genetic polymorphisms in the *AR* that may affect the AR activity and thus circulating androgen levels. Interestingly, two SNPs (rs5965433 and rs5919392) were found to be significantly associated with FT, with the first also borderline significantly associated with TT. However, it is noteworthy that these associations did not remained significant after Bonferroni correction. Two recent genome-wide association studies [30], [31] have identified several SNPs at different loci that were associated with serum T levels in middle-aged and elderly men. However, the *AR* gene was not described in these studies. Considering our relatively limited sample size, we suggest that analysis of our SNPs in those larger study populations may be required to confirm our findings.

Genetic variation in the *AR* gene influences circulating androgen levels, but may also affect body composition, muscularity or anthropometrics. Data on the association between CAG repeat length and muscle mass is limited and has been contradictory [8], [32], [33]. In our study, we could not find any relationship of CAG, GGN repeat length or the analysed SNPs in the *AR* with either body composition or measurements of muscularity. This might indicate that the relation of T with the muscle CSA is not related to genetic factors influencing androgen sensitivity, most likely because lower androgen sensitivity is compensated by elevated T levels.

Interestingly, we found that arm span and the 2D:4D finger length ratio were inversely associated with the number of CAG repeats, but not with the GGN repeat lenght or the analysed SNPs. The 2D:4D finger length ratio has been proposed as a marker of prenatal androgen action and of sensitivity to T, with a lower 2D:4D being associated with high androgen exposure [34], [35]. Given the hypothesis that elevated T levels in men with lower androgen sensitivity do not necessary show differences in androgen action, we can speculate that the negative effects on arm span and finger length might be mediated by the higher levels of FE_2_ levels found in men with longer CAG repeat length, as suggested by Huhtamieni IT *et al.* [36]. As most E_2_ produced in normal men is formed by aromatization of androgens [37], the higher T substrate availability in men with lower androgen sensitivity can explain the higher serum E_2_ levels. E_2_ is considered to be the main sex steroid involved in the development and maintenance of bone mass [18]. In addition, it is also important to initiate epiphyseal closure of long bones [38]. Therefore, we speculate that the presence of higher levels of E_2_ in men with lower androgen sensitivity, but preserved estrogen action, resulted in earlier termination of longitudinal bone growth during puberty, an event wich is clearly observed in boys with aromatase excess syndrome or familiar hyperestrogenism [39], [40].

To date, several studies have examined the possible relation of adult sex hormone concentrations [41], [42] and *AR* CAG number [43]–[45] with 2D:4D, but results are controversial. To our knowledge, there is only one study that has examined the relationship between GGN repeat variation in the *AR* and 2D:4D ratios [46], but no reports on the relationship between SNPs in *AR* and 2D:4D ratios exist.

The higher serum E_2_ levels found in men with a higher CAG repeat number might also play a direct role on muscle force since the negative association between E_2_ and grip strength and biceps force, and between FE_2_ and grip strength and biceps force in our study persisted after adjustment for T. Also Auyeung *et al.* [47] reported that E_2_ levels, though positively related to muscle mass, were negatively related to muscle strength. However, it should be noted that the participants of the latter study were much older, with lower T levels.

Possible effects of E_2_ on the regulation of muscle mass and function are still poorly understood. As skeletal muscle myoblasts and mature fibers express functional estrogen receptors (ER), a direct effect of E_2_ in muscle cells may occur [48], [49]. Although some studies have shown that E_2_ is involved in muscle recovery [50], [51] and has anabolic effects [52], [53], a negative role of E_2_ on the musculature has also been suggested by others. Several studies observed a decrease in muscle mass and force after E_2_ administration of ovariectomized rats [54]–[57], and Brown M *et al.* [50] found an increase in muscle mass and function in *ER* knockout mice. However, the exact mechanism by which estrogens regulate muscle mass still has to be elucidated.

We recognize that our study has some limitations. First, our study may have been limited by the relatively small sample size, by which small but significant associations might have been missed, especially for the genetics analysis. Secondly, observations within brothers are not completely independent from each other. However, all analyses in this study were performed using linear mixed-effects modelling with random intercepts to account for this interdependence. Furthermore, the cross-sectional design of this study does not allow us to draw conclusions on causality.

A major strength of this study is that we have used a highly precise LC-MS/MS method to determine T and E_2_ serum concentrations. Most other studies used direct immunoassays, which are thought to have a reduced specificity at lower concentrations, especially those for serum E_2_ [58], [59], which could explain some of the conflicting results reported. Also, our cohort of healthy men in a well-defined age range may have strengthened our results.

In summary, in this study we showed that age, physical activity, body composition, sex steroid levels and anthropometrics are all determinants of muscle mass and function in young men. Although the number of CAG repeats were related to sex steriod levels and anthropometrics, we have no evidence that variations in the *AR* gene also contributes to the between subject variation in muscle mass or muscle function in young healthy men.
